# What, when, and how food and beverage are advertised on Ghanian television

**DOI:** 10.1371/journal.pone.0325730

**Published:** 2025-06-09

**Authors:** Morgan Boncyk, Krystal K. Rampalli, Marian N. Winters, Muskaan K. Makkar, Silver Nanema, Gideon S. Amevinya, Amos Laar, Edward A. Frongillo, Christine E. Blake

**Affiliations:** 1 Department of Health Promotion, Education, and Behavior, University of South Carolina, South Carolina, United States of America; 2 Department of Population, Family & Reproductive Health, School of Public Health, University of Ghana, Accra, Ghana; Paris School of Business, FRANCE

## Abstract

Food marketing has increased volume, precision, and reach to influence viewers’ food attitudes, beliefs, and eating behaviors. What and how much people eat has implications for health. While many countries regulate food advertising to protect consumers and encourage healthy eating, Ghana has none. Understanding the content and framing of food and beverage advertisements can inform the development of effective policies and practices that encourage healthier diets. This content analysis examines the foods and beverages advertised, their timing, and marketing techniques on Ghanaian television. From February to May 2020, 486 hours of advertisements were recorded. Advertisements with ≥1 actors were coded for food type, actor characteristics (i.e., body size, gender, age, race), and marketing techniques (i.e., promotional characters, premium offers, goal frames). A total of 607 advertisements with 2,043 actors were analyzed. Two-thirds (66.8%) promoted foods categorized as unhealthy. Sugar-sweetened beverages (22.6%) were most frequent, followed by grains high in sugar and low in fiber (13.2%), recipe additions (13.1%), and supplements (10.2%). Half (52.9%) of advertisements used persuasive marketing strategies. Most actors were classified as underweight (72.1% v. 20.5% normal weight, 7.4% overweight/obese) with a balanced gender distribution (49.1% female). Most advertisements aired during evenings (37.7%) and weekdays (69.5%). Morning advertisements promoted more healthy foods, whereas evening and night advertisements promoted more unhealthy foods. Gain goal frames were most common for healthy foods (p < 0.001), hedonic frames for unhealthy foods (p < 0.001), and normative frames showed no difference (p = 0.54). Underweight actors frequently appeared in unhealthy advertisements (68.3% v. 56.0% normal weight, 59.0% overweight/obese), whereas normal-weight (44.0%) and overweight/obese actors (41.0% v. 31.7% underweight) appeared in healthy advertisements. Persuasive marketing strategies were frequently advertised with unhealthy foods (59.9%) and overweight/obese (54.9%) and male actors (53.6%). This study highlights the need for effective policies to regulate food marketing, promoting healthier diets and realistic body expectations.

## Introduction

The number of marketing strategies and the amount of money spent on marketing have steadily increased year after year as companies leverage mass media to shape perceptions and expectations by influencing consumers’ awareness and interpretation of specific information. Billions of dollars are spent each year on mass media, with food and beverage companies among the leading spenders. Television advertisements are a common medium for food and beverage marketing, defined as any form of commercial communication or message to increase the recognition, appeal, and/or consumption of a product or service [1]. McCombs and Shaw’s Agenda Setting Theory and Goffman and Entman’s Framing Theory explain how mass media, such as television advertisements, influences consumers’ awareness and interpretation of specific information, either positively or negatively [2–4]. These theories illustrate how repeated and continuous exposure to television advertisements can shape viewers’ perceptions and attitudes toward the advertised food, potentially shifting sociocultural norms and expectations [5,6].

Continuous exposure to food advertisements can influence viewers’ behavior, leading to increased consumption of the food advertised among children [7] and adults [8]. Exposure to food advertisements is problematic, as most food and beverage television advertisements use persuasive marketing techniques to promote unhealthy foods [9]. Unhealthy foods already make up a substantial proportion of total dietary intake, and their consumption is projected to increase with globalization, increasing the availability and accessibility of unhealthy foods, and advancements in digital technology, increasing the precision and reach of advertisements. Television viewership in Ghana is high; 72% and 92% of children (4–8 years) exceeded two hours of view time per day on weekdays and weekends, respectively, in South Ghana in 2021 [10]. Among adults (15–49 years), 62% watched television weekly in 2022, higher among the educated, wealthy, and urban residents [11]. Over time, this increased exposure and consumption of unhealthy foods is expected to contribute to excessive caloric intake and diet-related non-communicable diseases (NCDs) [12,13].

Diet-related NCDs are the leading cause of worldwide morbidity and mortality, threatening the attainment of the 2030 Sustainable Development Goals [14–16]. To address NCD risk and improve population health, many countries have developed food-based dietary guidelines, advertisement regulations, and policies to limit consumers’ access to and consumption of sugar-sweetened beverages and foods high in salt, added sugars, and saturated and trans fatty acids [17–19]. In Ghana, longstanding undernutrition, micronutrient deficiency, and malnutrition, combined with the recent increased availability of highly processed foods high in energy, saturated or trans fats, sodium, and added sugars, have hindered the achievement of global nutrition targets and contribute to the increase in diet-related NCDs [15,20]. Ghana only recently implemented food-based dietary guidelines [17,18] and alcohol-based food-marketing policies [17–19], but do not have guidelines, regulations, or policies for food advertisements, which have effectively restricted unhealthy food advertisements in other countries globally [21–23]. Further, there is limited information for program and policy planners on the types of foods and beverages and marketing strategies used for promotion of these products in Ghana. A better understanding of the content of current food and beverage advertisements is needed to inform the development of similar regulations in Ghana.

Globalization has increased not only access to highly processed foods but also introduced new cultural ideas and expectations for physical appearance, shifting sociocultural norms, particularly among younger generations. Younger generations in Africa are navigating between traditional beauty ideals that associate larger female body sizes with dignity, health, fertility, beauty, and wealth [24–32] and Western ideals that prefer thinner bodies, historically associated with poverty and disease [32,33]. Bandura’s Social Learning Theory explains how we observe others and model those we deem influential to our social spheres as we aspire to be more like them [34,35]. This theory helps to explain how promotional characters, the most prominent driver of body aspirations [36], tap into individual desires to model the behaviors of those to whom we look up [34], potentially influencing food consumption and body aspirations. Advertisements that attempt to link consumption of avoidance of certain foods with attractiveness, risk promoting unhealthy aspirations. Most food marketing policies designed to protect children focus on things like the use of cartoon characters to promote junk food marketing to small children but to date have not addressed the influence of actors unhealthy of unrealistic physical characteristics to increase the appeal and consumption of unhealthy foods [37]. A better understanding of the content of food advertisements in Ghana can aid in development of future food advertising policies and regulations.

In response to marketing tactics, many countries have implemented guidelines to restrict the advertisement of unhealthy foods during children’s peak viewing hours [7,38]; however, efforts to reduce these advertisements targeting adults have been limited. There is a lack of evidence on what foods are promoted in mass media, the temporal orientation of when healthy and unhealthy foods appear, and how marketing strategies are used to promote healthy and unhealthy foods. Such information is important for the development of effective policies and practices that encourage healthier diets for the entire population. This study aims to provide a comprehensive overview of what, when, and how foods and beverages are advertised on Ghanaian television with an emphasis on the way physical appearance is used to market healthy and unhealthy foods. We achieved this aim by examining what foods were advertised, when they are advertised, and how they are advertised, including assessment of the physical characteristics of actors, to inform policies and practices to regulate mass media with the intent of increasing healthy food consumption.

## Methods

### Study design

The study used a content analysis of television food and beverage advertisements in Ghana following the International Network for Food and Obesity/non-communicable diseases (NCDs) Research, Monitoring and Action Support (INFORMAS) protocol [39]. INFORMAS aimed to systematically and consistently monitor the extent of unhealthy food promotions on television advertisements within and across countries and over time to estimate exposure levels to inform policy development and government accountability. The project entitled Measurement, Evaluation, Accountability and Leadership Support for NCDs Prevention (MEALS4NCDs) aimed to describe the nature and extent of unhealthy foods and beverage advertised on Ghanaian television; more details can be found elsewhere [20].

### Data collection

Television advertisements were manually recorded with a DVD recorder at the time of broadcast from February to May 2020. With consultation from Ghanaian media industry experts, the top three of 51 free-to-air local/national television channels with broadcast advertisements were identified based on child viewership (Adom TV, TV3, UTV). Live programming on these channels was simultaneously video recorded over nine days (five weekdays and four weekend days) directly onto DVD’s using standalone DVD-R player/recorders. DVD recordings were then saved as MP4 files for digital playback using desktop computers. A composite sampling method was used to select a representative broadcast period, recording days that covered a school term (three months) and vacation month, excluding national holidays and special events. Recording covered 18 hours a day (6:00 am to 11:59 pm) for nine days over three channels, totalling 486 hours. This was above what was considered the minimum amount of time (i.e., 18 hours a day for eight days) to obtain estimates established by the multi-country comparison protocol for television advertisements established by INFORMAS. Further details of the sampling procedures are presented elsewhere [20,39]. Television advertisements are spot advertisements that appear during a broadcast or between programs, excluding commercial activities such as product placement or sponsorship of television shows. Advertisements were included when a food or beverage appeared in the advertisements, regardless of whether it was the focal point of the advertisement. Advertisements were excluded if they did not contain at least one full-body view of an actor. These advertisements were not unique, as they could have appeared multiple times. Ethical approval was received from the University of Ghana Institute of Statistical, Social and Economic Research’s for the Humanities (128/19–20) and the University of South Carolina (PRO00097113).

### Instruments

Data were analyzed as per the INFORMAS protocol using standard content analysis procedures [39,40]. An *a priori* codebook (S1 Table) was developed based interviews with adolescents in Ghana about body image and food choice [41], contextual observations of Ghana’s food environment, and common themes reported in the literature on body image, West Africa, nutrition transition, food choices, and food and beverage marketing. A primary coder (KKR) developed the codebook and trained two secondary coders (SN, GSA) on its application using definitions and sample advertisements as examples. Training for classifying actor characteristics occurred with various types of marketing, such as magazines. This codebook was tested on 10% of the data before applied by three coders for the remaining data. Coders entered the data directly into Microsoft Excel while simultaneously watching the DVD with the advertisement. Only advertisements with food and/or beverage marketing were coded. The first five actors to appear per advertisement were recorded.

### Measures

The content analysis coded television advertisements for the following characteristics: the brand and product, channel, time and day aired, types of foods and beverages advertised, the actor characteristics (i.e., body size, gender, age, race), and the persuasive marketing technique used (i.e., goal frames, promotional character, premium offer). To assess inter-coder reliability, the three coders all coded the same content applying the code book to one full day of TV recordings (109 advertisements/ 18% of the total sample). Inter-coder agreement using this code book was > 99, demonstrating excellent reliability. The remaining advertisements were coded by a single coder.

#### What foods and beverages.

The food and beverages shown in each advertisement were categorized as either healthy or unhealthy following a modified version of the INFORMAS country-specific food categorization system, based on dietary guidelines from various countries to allow for comparability across countries [39]. Ingredients and nutrition information about specific food products was obtained through review of food labels and food composition information and publicly available product information provided online through company websites. Foods categorized as miscellaneous in the INFOMRAS classification system were individually assessed by a dietician experienced in diet and nutrition in Ghana (CEB) to determine whether they belong in the healthy or unhealthy category. Healthy included INFORMAS core foods and beverages: high-fiber cereals, plain breads, rice, and noodles without added fat, sugar or salt; fruits and vegetables without added fats, sugars or salt; low-fat milks, yoghurts, cheese and dairy alternatives; protein-rich meats and meat alternatives; baby food excluding milk formulae; unflavored mineral water and soda waters; and vitamin or mineral supplements and sugar-free chewing gum. Unhealthy included INFORMAS non-core foods and beverages: high sugar, low-fiber breakfast cereals and breads, flavored or fried instant rice and noodles; sweet and savory snacks, chocolate, and candy; high-fat milks, yoghurts, and cheese, ice cream, iced confection and desserts; processed or preserved and ready to eat meats; sugar-sweetened beverages, including juices; alcohol; recipe additions (bouillon cubes, oil, dried herbs and seasoning); and high-fat pastes, sauces, butters and animal fats, high fat savory sauces and soups.

#### When aired.

Timing of advertisements were categorized by their start time into morning (6:00–11:59 am), afternoon (12:00–4:59 pm), evening (5:00–8:59 pm), and night (9:00 pm to 12:00 am). Day of week the advertisement was aired was recorded, with Saturday and Sunday considered the weekend.

#### How advertised.

Advertisements were coded for their marketing techniques and actor characteristics. Persuasive marketing techniques included promotional characters (i.e., celebrities or famous sportsperson, amateur sportsperson, appealed to children, cartoon) and premium offers (i.e., gift, price discount, charity). Advertisement’s goal frames, how they appeal to the way people process and act on information, include hedonic (improving the way one feels), gain (benefit personal resources), and normative (behave appropriately) [42]. Actor characteristics (i.e., body size, race, age, gender) were identified based on the coder’s perception. Body size was categorized according to Ettarh’s silhouettes, comprising 18 sizes ranging from very thin to very obese [43]. These silhouettes have been validated against objective body size measures to classify individuals as underweight (silhouettes 1–5), normal weight (silhouettes 6–9), overweight (silhouettes 10–13), or obese (silhouettes 14–18) [44,45]. Inter-rater reliability of body sizes was 94% agreement in body size using ±1 for the 18 body sizes. Overweight and obese were combined for analysis due to small samples. Race included Black, White, Asian, and indeterminant when the race could not be determined. Age was grouped as child (≤11 years), adolescent (12–17 years), or adult (≥18 years). Gender included male or female. Coders recorded the total number of actors in each characteristic category for the first five actors in each advertisement.

### Data analysis

Data analysis followed a pre-specified analytic plan to align with the study aim and research questions, ensuring the data analysis was structured to provide comprehensive insights into the characteristics of actors portrayed in Ghana television food advertisements. Two advertisements were excluded from the analysis due to coding discrepancies in the assignment of actors’ gender. Descriptive statistics, including frequencies and percentages, were computed by advertisements and weighted by the number of actors in each advertisement to summarize the type of foods and beverages, actor characteristics, and marketing techniques. Bivariate analyses were conducted for mutually exclusive group comparisons using Pearson’s chi-square test. Comparative analysis for healthy and unhealthy foods excluded advertisements that included multiple categories. Analysis of descriptive and bivariate statistics was performed in Stata18 [46].

## Results

The sample had a total of 607 advertisements, with 20% occurring during soap operas and 20% during news programs. A third (33.2%) of advertisements were for healthy and two-thirds (66.8%) unhealthy foods and beverages (Table 1). Advertisements most frequently included sugar-sweetened beverages (22.6%), unhealthy grains low in fat, sugar, salt, and high in fiber (13.2%), recipe additions (e.g., soup cubes, oil, seasoning) (13.1%), supplements (10.2%), and alcohol (9.9%). Healthy (52.2 ± 15.5 seconds) and unhealthy (49.8 ± 16.4 seconds) advertisements had similar durations.

**Table 1:** Time of day and day of week food and beverage were aired during Ghana’s television advertisements.

n = ads; n = actors Data reporter as % (n=)		Total (n = 567; n = 1,964)	Time of Day^a^					Day of Week^b^		
			Morning (n = 95; n = 301)	Afternoon (n = 179; n = 583)	Evening (n = 207; n = 760)	Night (n = 86; n = 320)	p-value	Weekday (n = 407; n = 1,467)	Weekend (n = 160; n = 497)	p-value
**Healthy**										
Supplements	ads	10.2 (58)	14.7 (14)	9.5 (17)	7.7 (16)	12.8 (11)	0.24	11.5 (47)	6.9 (11)	0.098
	actors	7.4 (145)	12.0 (36)	7.2 (42)	5.5 (42)	7.8 (25)	0.004	8.1 (119)	5.2 (26)	0.034
Grain (low fat/sugar /salt, high fiber)	ads	9.0 (51)	9.5 (9)	10.1 (18)	9.2 (19)	5.8 (5)	0.72	10.1 (41)	6.2 (10)	0.15
	actors	9.0 (177)	10.0 (30)	11.3 (66)	8.6 (65)	5.0 (16)	0.014	10.2 (150)	5.4 (27)	0.001
Dairy (low-fat)	ads	7.2 (41)	9.5 (9)	7.3 (13)	5.3 (11)	9.3 (8)	0.49	6.4 (26)	9.4 (15)	0.22
	actors	8.3 (163)	11.6 (35)	8.9 (52)	5.8 (44)	10.0 (32)	0.007	7.0 (103)	12.1 (60)	<0.001
Baby food	ads	3.0 (17)	4.2 (4)	2.2 (4)	4.3 (9)	0.0 (0)	0.19	3.4 (14)	1.9 (3)	0.33
	actors	1.7 (34)	2.7 (8)	1.4 (8)	2.4 (18)	0.0 (0)	0.024	1.9 (28)	1.2 (6)	0.30
Fruit and vegetable	ads	0.7 (4)	1.1 (1)	0.0 (0)	1.4 (3)	0.0 (0)	0.30	0.7 (3)	0.6 (1)	0.89
	actors	0.8 (16)	1.7 (5)	0.0 (0)	1.4 (11)	0.0 (0)	0.003	1.0 (15)	0.2 (1)	0.078
Water	ads	1.9 (11)	0.0 (0)	2.8 (5)	1.9 (4)	2.3 (2)	0.45	1.7 (7)	2.5 (4)	0.54
	actors	1.5 (29)	0.0 (0)	1.9 (11)	2.1 (16)	0.6 (2)	0.031	0.9 (13)	3.2 (16)	<0.001
Meat	ads	1.8 (10)	5.3 (5)	0.0 (0)	2.4 (5)	0.0 (0)	0.007	1.5 (6)	2.5 (4)	0.40
	actors	1.3 (26)	4.3 (13)	0.0 (0)	1.7 (13)	0.0 (0)	<0.001	1.2 (18)	1.6 (8)	0.52
Supermarket	ads	0.4 (2)	1.1 (1)	0.0 (0)	0.5 (1)	0.0 (0)	0.50	0.5 (2)	0.0 (0)	0.37
	actors	0.5 (10)	1.7 (5)	0.0 (0)	0.7 (5)	0.0 (0)	0.005	0.7 (10)	0.0 (0)	0.065
**Unhealthy**										
SSBs and juice	ads	22.6 (128)	30.5 (29)	22.9 (41)	22.7 (47)	12.8 (11)	0.043	24.8 (101)	16.9 (27)	0.042
	actors	24.8 (488)	32.2 (97)	27.8 (162)	25.8 (196)	10.3 (33)	<0.001	28.3 (415)	14.7 (73)	<0.001
Grain (high sugar, low fiber)	ads	13.2 (75)	5.3 (5)	22.9 (41)	12.6 (26)	3.5 (3)	<0.001	9.1 (37)	23.8 (38)	<0.001
	actors	14.6 (286)	6.6 (20)	25.9 (151)	13.2 (100)	4.7 (15)	<0.001	10.0 (146)	28.2 (140)	<0.001
Recipe additions (soup cubes, oil, seasoning)	ads	13.1 (74)	13.7 (13)	16.8 (30)	10.1 (21)	11.6 (10)	0.27	11.1 (45)	18.1 (29)	0.025
	actors	10.7 (210)	12.3 (37)	12.0 (70)	10.0 (76)	8.4 (27)	0.27	8.7 (127)	16.7 (83)	<0.001
Alcohol	ads	9.9 (56)	0.0 (0)	0.0 (0)	11.1 (23)	38.4 (33)	<0.001	12.3 (50)	3.8 (6)	0.002
	actors	12.9 (254)	0.0 (0)	0.0 (0)	13.0 (99)	48.4 (155)	<0.001	15.5 (228)	5.2 (26)	<0.001
Dairy (high fat/salt)	ads	5.8 (33)	4.2 (4)	3.9 (7)	9.7 (20)	2.3 (2)	0.028	6.1 (25)	5.0 (8)	0.60
	actors	5.7 (112)	4.3 (13)	2.6 (15)	9.7 (74)	3.1 (10)	<0.001	6.5 (95)	3.4 (17)	0.011
Processed meat	ads	0.9 (5)	1.1 (1)	0.6 (1)	1.4 (3)	0.0 (0)	0.62	1.2 (5)	0.0 (0)	0.16
	actors	0.5 (10)	0.7 (2)	0.3 (2)	0.8 (6)	0.0 (0)	0.35	0.7 (10)	0.0 (0)	0.065
Snacks and candy	ads	0.7 (4)	3.2 (3)	0.0 (0)	0.5 (1)	0.0 (0)	0.017	0.7 (3)	0.6 (1)	0.89
	actors	1.0 (20)	5.0 (15)	0.0 (0)	0.7 (5)	0.0 (0)	<0.001	1.0 (15)	1.0 (5)	0.97
Pastes, sauces, and butter	ads	0.7 (4)	0.0 (0)	1.1 (2)	0.5 (1)	1.2 (1)	0.68	0.2 (1)	1.9 (3)	0.037
	actors	0.7 (14)	0.0 (0)	0.7 (4)	0.7 (5)	1.6 (5)	0.14	0.3 (5)	1.8 (9)	<0.001

^a^Morning was considered 6:00–11:59 am, afternoon 12:00–4:59 pm, evening 5:00–8:59 pm, and night 9:00 pm to 12:00 am.

^b^Saturday and Sunday were considered the weekend. Type of food advertised is not mutually exclusive. Excluded advertisements that promoted healthy and unhealthy foods and beverages. Healthy grains included high-fiber cereals, plain breads, rice, and noodles without added fat, sugar or salt; Supplements included vitamin/mineral and sugar-free chewing gum; Healthy dairy included low-fat milks, yoghurts, cheese and dairy alternatives; Baby food excluded milk formulae; Water included unflavored mineral and soda waters; Healthy meats included meat and meat alternatives; Fruits and vegetables included fruits/vegetable without added fats, sugars or salt; Supermarkets advertised healthy food in front of storefronts; Sugar-sweetened beverages (SSBs) included sugar sweetened drinks and fruit juice/drinks; Unhealthy grains included high sugar, low-fiber breakfast cereals and breads, flavored/fried instant rice and noodles; Recipe additions included soup cubes, oils, dried herbs and seasonings; Unhealthy dairy included high-fat milks, yoghurts, and cheese, ice cream, iced confection and desserts; Unhealthy meats included processed or preserved and ready to eat meats; Unhealthy snacks included sweet and savory snack foods, chocolate and candy; Other high fat/salt foods included meat/fish/bean pastes, XO sauce, butter and animal fats, high fat savory sauces and soups

Most advertisements aired during evenings (37.7%) and weekdays (69.5%) (Table 1). Foods were advertised using persuasive marketing techniques and with various goal frames (Table 2). Half (49.6%) of advertisements used promotional characters, such as celebrities or famous sportsperson (34.1%), or appealed to children (10.9%), while premium offers were rarely used (5.8%). Advertisements equally used gain (45.1%), hedonic (42.8%), and normative (39.7%) goal frames.

**Table 2:** How persuasive marketing techniques and goal frames were used in Ghana’s television advertisements for healthy and unhealthy food and beverages and by actors’ body size and gender.

n = ads; n = actors Data reporter as % (n=)		Total (n = 607; n = 2,043)	Type of Food^a^			Body Size^b^			Gender^b^	
			Healthy (n = 188; n = 570)	Unhealthy (n = 379; n = 1,394)	p-value	Underweight (n = 542; n = 1,472)	Normal Weight (n = 304; n = 419)	Overweight/ Obese (n = 133; n = 152)	Male (n = 526; n = 1,039)	Female (n = 509; n = 1,004)
**Persuasive marketing technique** ^c^										
Any techniques	ads	52.9 (321)	31.9 (60)	59.9 (227)	<0.001	48.3 (262)	51.0 (155)	54.9 (73)	53.6 (282)	47.3 (241)
	actors	50.6 (1,033)	27.4 (156)	59.3 (827)	<0.001	51.3 (755)	45.1 (189)	58.6 (89)	58.3 (606)	42.5 (427)
Promotional character^b^	ads	49.6 (301)	31.9 (60)	55.1 (209)	<0.001	45.0 (244)	50.0 (152)	41.4 (55)	49.8 (262)	43.4 (221)
	actors	45.7 (933)	27.4 (156)	52.9 (737)	<0.001	47.4 (697)	42.7 (179)	37.5 (57)	53.4 (555)	37.6 (378)
Celebrity^d^	ads	34.1 (207)	14.9 (28)	38.8 (147)	<0.001	29.3 (159)	35.2 (107)	28.6 (38)	34.0 (179)	29.7 (151)
	actors	30.3 (620)	16.8 (96)	34.7 (484)	<0.001	30.5 (449)	31.3 (131)	26.3 (40)	33.7 (350)	26.9 (270)
Amateur Sportsperson	ads	10.2 (62)	0.0 (0)	16.4 (62)	<0.001	11.4 (62)	1.6 (5)	9.8 (13)	11.4 (60)	9.6 (49)
	actors	14.6 (298)	0.0 (0)	21.4 (298)	<0.001	19.0 (280)	1.2 (5)	8.6 (13)	23.1 (240)	5.8 (58)
Appealed to children	ads	10.9 (66)	17.0 (32)	9.0 (34)	0.005	10.5 (57)	14.1 (43)	3.0 (4)	10.8 (57)	10.8 (55)
	actors	8.8 (179)	10.5 (60)	8.5 (119)	0.16	8.8 (129)	11.0 (46)	2.6 (4)	8.9 (92)	8.7 (87)
Cartoon	ads	6.8 (41)	9.0 (17)	6.3 (24)	0.24	6.6 (36)	7.2 (22)	0.0 (0)	6.8 (36)	8.1 (41)
	actors	5.6 (115)	6.0 (34)	5.8 (81)	0.89	6.3 (93)	5.3 (22)	0.0 (0)	5.3 (55)	6.0 (60)
Premium offer^b^	ads	5.8 (35)	0.0 (0)	8.2 (31)	<0.001	6.1 (33)	1.0 (3)	13.5 (18)	6.7 (35)	6.5 (33)
	actors	8.0 (164)	0.0 (0)	10.9 (152)	<0.001	8.3 (122)	2.4 (10)	21.1 (32)	9.5 (99)	6.5 (65)
Gift	ads	4.6 (28)	0.0 (0)	7.4 (28)	<0.001	4.8 (26)	1.0 (3)	13.5 (18)	5.3 (28)	5.5 (28)
	actors	6.9 (140)	0.0 (0)	10.0 (140)	<0.001	6.7 (98)	2.4 (10)	21.1 (32)	8.6 (89)	5.1 (51)
Price discount	ads	2.0 (12)	0.0 (0)	2.6 (10)	0.025	2.2 (12)	0.0 (0)	5.3 (7)	2.3 (12)	2.4 (12)
	actors	2.8 (57)	0.0 (0)	3.4 (47)	<0.001	2.4 (36)	0.0 (0)	13.8 (21)	2.1 (22)	3.5 (35)
Charity	ads	0.3 (2)	0.0 (0)	0.0 (0)	--	0.4 (2)	0.0 (0)	0.0 (0)	0.4 (2)	0.0 (0)
	actors	0.1 (2)	0.0 (0)	0.0 (0)	--	0.1 (2)	0.0 (0)	0.0 (0)	0.2 (2)	0.0 (0)
**Goal Frame** ^b^										
Normative	ads	39.7 (241)	35.6 (67)	38.3 (145)	0.54	38.2 (207)	59.5 (181)	43.6 (58)	42.4 (223)	43.4 (221)
	actors	43.5 (889)	44.0 (251)	40.9 (570)	0.20	38.1 (561)	60.9 (255)	48.0 (73)	36.1 (375)	51.2 (514)
Gain	ads	45.1 (274)	73.4 (138)	35.6 (135)	<0.001	47.2 (256)	41.8 (127)	51.9 (69)	44.1 (232)	49.1 (250)
	actors	43.2 (883)	67.0 (382)	35.7 (497)	<0.001	45.8 (674)	32.9 (138)	46.7 (71)	46.5 (483)	39.8 (400)
Hedonic	ads	42.8 (260)	22.9 (43)	49.9 (189)	<0.001	40.8 (221)	38.2 (116)	38.3 (51)	44.1 (232)	36.9 (188)
	actors	42.0 (858)	27.5 (157)	48.3 (673)	<0.001	43.1 (635)	40.6 (170)	34.9 (53)	44.4 (461)	39.5 (397)

^a^Healthy foods were considered foods and beverages high in fiber and low in sugar, salt, and fats. Healthy and unhealthy advertisements exclude those that promoted healthy and unhealthy;

^b^Indicates that categories were not mutually exclusive;

^c^Persuasive marketing technique indicates that at least one promotional character or premium offer were used;

^d^Celebrities included famous sportspersons.

**Fig 1:**
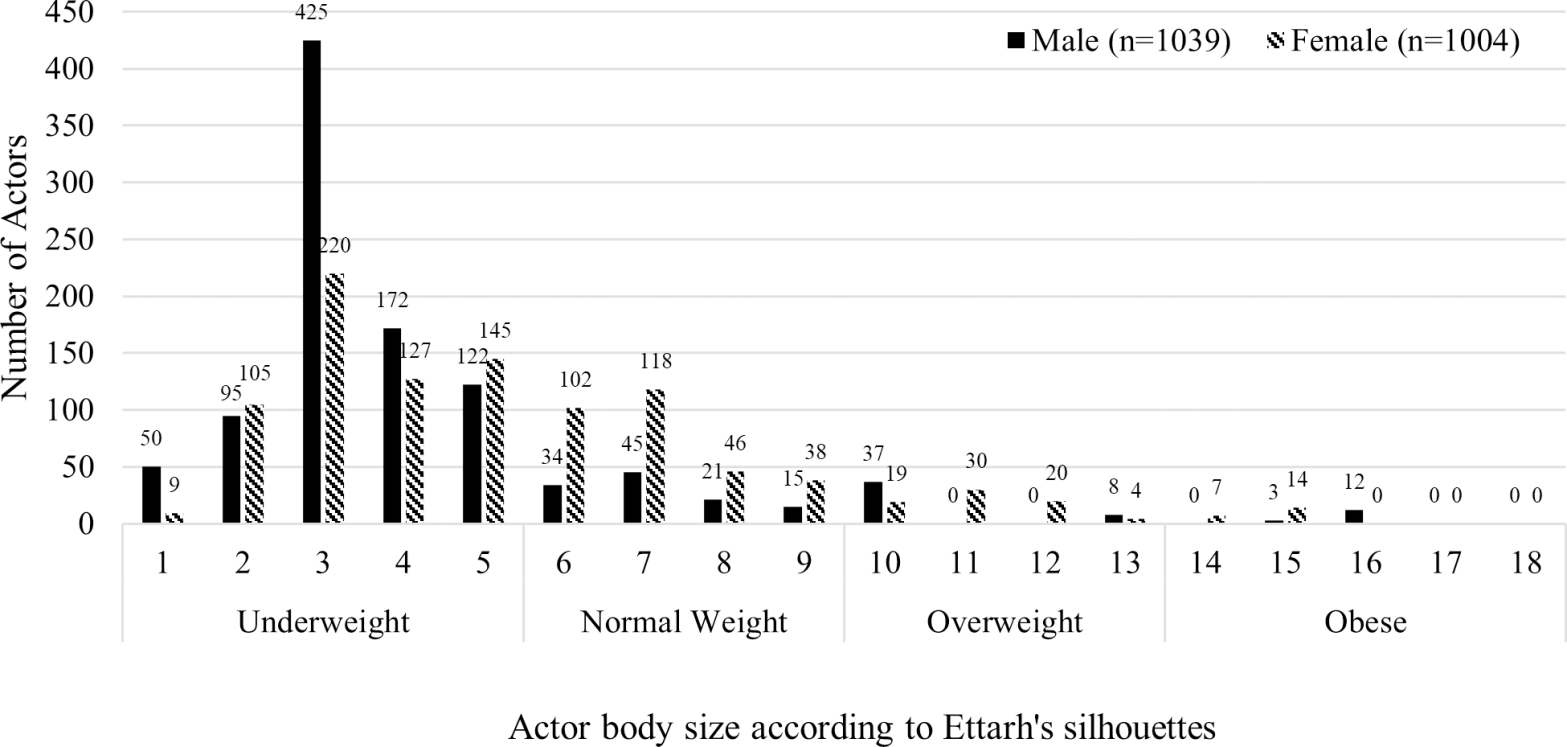
Actor’s body size, according to Ettarh’s silhouettes [43], by gender portrayed across 607 food and beverage television advertisements in Ghana.

Actors (n = 2,043) were evenly distributed by gender (50.9% male) but not by body size. Three-quarters (72.1%) of the actors advertising these foods were underweight, 20.5% normal weight, and 7.4% overweight/obese (Fig 1). Actors classified as underweight were majority male (58.8% male), whereas actors classified as normal weight and overweight/obese were majority female (73.0% and 61.8%, respectively). Half (49.9%) of advertisements portrayed child actors, a quarter (25.0%) portrayed adolescents, and all (100%) portrayed adult actors. Actors were primarily Black (97.8%). Only a few were White (2.0%), and all White actors were underweight.

Most advertisements took place during evenings (37.7%) and weekdays (69.5%) (Table 1). Time of day and day of week were found to be associated with the airing of unhealthy, but not healthy, food and beverage advertisements: sugar-sweetened beverages were advertised more in the morning (30.5%, p = 0.043) and on weekdays (24.8%, p = 0.042), unhealthy grains in the afternoon (22.9%, p < 0.001) and on weekends (23.8%, p < 0.001), alcohol at night (38.4%, p < 0.001) and on weekdays (12.3%, p = 0.002), and recipe additions on weekends (23.8%, p = 0.25).

Advertisements for unhealthy foods more often used promotional characters (55.1% v. 31.9% healthy, p < 0.001), premium offers (8.2% v. 0% healthy, p < 0.001), and a hedonic frame (49.9% v. 22.9% healthy, p < 0.001), whereas advertisements for healthy foods more often used a gain frame (73.4% v. 35.6% unhealthy, p < 0.001) (Table 2).

**Fig 2:**
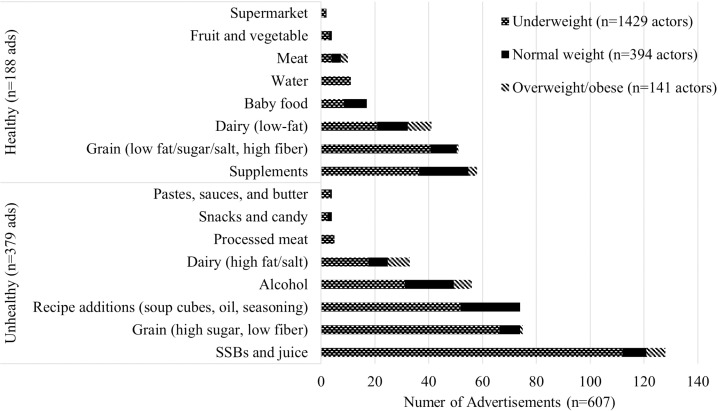
Foods promoted and the proportion of actors’ body sizes portrayed in Ghana’s food and beverage television advertisements. Excluded advertisements that promoted healthy and unhealthy foods and beverages; Healthy grains included high-fiber cereals, plain breads, rice, and noodles without added fat, sugar or salt; Supplements included vitamin/mineral and sugar-free chewing gum; Healthy dairy included low-fat milks, yoghurts, cheese and dairy alternatives; Baby food excluded milk formulae; Water included unflavored mineral and soda waters; Healthy meats included meat and meat alternatives; Fruits and vegetables included fruits/vegetable without added fats, sugars or salt; Supermarkets advertised healthy food in front of storefronts; Sugar-sweetened beverages (SSBs) included sugar sweetened drinks and fruit juice/drinks; Unhealthy grains included high sugar, low-fiber breakfast cereals and breads, flavored/fried instant rice and noodles; Recipe additions included soup cubes, oils, dried herbs and seasonings; Unhealthy dairy included high-fat milks, yoghurts, and cheese, ice cream, iced confection and desserts; Unhealthy meats included processed or preserved and ready to eat meats; Unhealthy snacks included sweet and savory snack foods, chocolate and candy; Other high fat/salt foods included meat/fish/bean pastes, XO sauce, butter and animal fats, high fat savory sauces and soups.

There were differences in body sizes, age, and gender of actors included in advertisements for healthy versus unhealthy foods and the time of day and day of week aired. Actors classified as underweight were more often included in unhealthy advertisements (74.3% v. 61.7% normal weight, 63.1% overweight/obese) (Fig 2). Children appeared more frequently in healthy food and beverage advertisements (55%) than in unhealthy ones (45%), whereas adolescents appeared more often in unhealthy (29%) versus healthy (23%) advertisements. Gender of actors in food and beverage advertising did not vary by type of food advertised. Advertisements with actors classified as underweight were more frequent in afternoons (31.5% v. 17.3% overweight/obese) (Table 3). Evenings and nights saw an increase in actors classified as overweight/obese (evening: 48.7% v. 39.7% underweight; night: 21.1% v. 12.6%). Males were more prevalent during the weekdays (77.5% v. 69.0% female). Male actors were more frequent in advertisements that included a promotional character (53.4% v. 37.6% females) (Table 2). Specifically, amateur sportspersons were more prevalent in advertisements that contained a male (23.1% v. 5.8% female) or underweight actor (19.0% v. 1.2% normal weight, 8.6% overweight/obese). Actors classified as overweight/obese appeared in more advertisements that contained a premium offer (21.1%; v. 8.3% underweight, 2.4% normal weight). Male actors were more frequent in gift offers (8.6% v. 5.1% female). Actors classified as underweight were evenly distributed across goal frames while actors classified as normal weight were most frequent in normative frames (60.9% v. 32.9% gain, 40.6% hedonic) and actors classified as overweight/obese were most frequent in gain (46.7%) and normative frames (48.0% v. 34.9% hedonic). Female actors were most frequent in normative frames (51.2% v. 39.8% gain, 39.5% hedonic), while male actors were most frequent in gain frames (46.5% v. 44.4% hedonic, 36.1% normative).

**Table 3:** Type of food and/or beverage advertised, time of day and day of week that actors’ body sizes and genders were aired during Ghana’s television food and beverage advertisements.

n = ads; n = actors Data reporter as % (n=)		Total (n = 607; n = 2,043)	Body Size^a^			Gender^a^	
			Underweight (n = 542; n = 1,472)	Normal Weight (n = 304; n = 419)	Overweight/ Obese (n = 133; n = 152)	Male (n = 526; n = 1,039)	Female (n = 509; n = 1,004)
**Type of food or beverage** ^a^ ^,^ ^b^							
Healthy	ads	33.2 (188)	31.7 (168)	44.0 (124)	41.0 (50)	31.8 (158)	32.6 (159)
	actors	29.0 (570)	25.7 (367)	38.3 (151)	36.9 (52)	28.7 (286)	29.4 (284)
Unhealthy	ads	66.8 (379)	68.3 (362)	56.0 (158)	59.0 (72)	68.2 (339)	67.4 (329)
	actors	71.0 (1,394)	74.3 (1,062)	61.7 (243)	63.1 (89)	71.3 (711)	70.6 (683)
**Time of day**							
Morning	ads	16.0 (97)	16.8 (91)	18.4 (56)	14.3 (19)	16.2 (85)	15.5 (79)
	actors	15.2 (311)	15.2 (224)	16.2 (68)	12.5 (19)	14.2 (148)	16.2 (163)
Afternoon	ads	32.0 (194)	31.5 (171)	28.6 (87)	17.3 (23)	31.6 (166)	31.4 (160)
	actors	30.2 (617)	32.4 (477)	27.0 (113)	17.8 (27)	31.3 (325)	29.1 (292)
Evening	ads	37.7 (229)	36.7 (199)	32.2 (98)	48.9 (65)	38.8 (204)	36.9 (188)
	actors	38.9 (794)	39.7 (585)	32.2 (135)	48.7 (74)	41.1 (427)	36.6 (367)
Night	ads	14.3 (87)	14.9 (81)	20.7 (63)	19.5 (26)	13.5 (71)	16.1 (82)
	actors	15.7 (321)	12.6 (186)	24.6 (103)	21.1 (32)	13.4 (139)	18.1 (182)
**Day of week**							
Weekday	ads	69.5 (422)	72.3 (392)	67.8 (206)	70.7 (94)	71.1 (374)	69.9 (356)
	actors	73.3 (1,498)	74.5 (1,097)	69.2 (290)	73.0 (111)	77.5 (805)	69.0 (693)
Weekend	ads	30.5 (185)	27.7 (150)	32.2 (98)	29.3 (39)	28.9 (152)	30.1 (153)
	actors	26.7 (545)	25.5 (375)	30.8 (129)	27.0 (41)	22.5 (234)	31.0 (311)

^a^Indicates that categories were not mutually exclusive;

^b^Healthy foods were considered foods and beverages high in fiber and low in sugar, salt, and fats. Healthy and unhealthy advertisements exclude those that promoted healthy and unhealthy, denominators: total (n = 567 advertisements, n = 1964 actors), underweight (n = 530 advertisements; n = 1429 actors), normal weight (n = 282 advertisements, n = 394 actors), overweight/obese (n = 122 advertisements, n = 141 actors), male (n = 497 advertisements, n = 997 actors), female (n = 488 advertisements, n = 967 actors).

## Discussion

Globalization and persuasive marketing of food and beverages influence consumption patterns and the appeal of advertised foods. We provide a comprehensive overview of what, when, and how foods and beverages are advertised in Ghana amidst a nutrition transition and shift in sociocultural norms to inform policies and practices to regulate mass media. A content analysis of 486 hours of food and beverage advertisements was conducted across three popular television channels, identifying 607 advertisements featuring a full-body view of 2,043 actors. Most advertisements contained unhealthy foods and persuasive marketing techniques, and actors were classified as underweight with Ettarh silhouettes. Unhealthy advertisements were more frequent during nights, evenings, and weekends, with persuasive marketing techniques, and included more actors classified as underweight, whereas healthy foods were more frequent in the morning and with actors classified as normal weight or overweight/obese.

Two advertisements for foods and beverages high in sugar, salt, and fat appeared for every nutrient-rich, minimally processed food advertisement. The prevalence of unhealthy food and beverage advertisements is concerning, given the rising rates of diet-related NCDs and obesity. While many foods classified as unhealthy in this analysis contain essential nutrients and are often fortified with micronutrients, these foods should be consumed in moderation to reduce diet-related NCDs. As with food and beverage advertisements worldwide [47], most food and beverage marketing occurs from a few transnational companies that are considered the driving force behind food consumption high in sugar, salt, and fat [48,49]. To mitigate unhealthy food advertisements and rising rates of NCDs, more healthy foods should be advertised, which can be done by regulating airtime for unhealthy foods or subsidizing airtime for healthy foods. Ludwig and Nestle explain how the food industry should be held to the same standard as the car industry in that companies that sell cars harmful to the environment are required to pay higher taxes and comply with regulations and mandates [50]. Food companies should be held to the same accountability for their impact on the population’s health [50]. Preventing NCDs through adopting healthy food choices is essential for ensuring the health and well-being of the population [48,51]. The disproportional promotion of unhealthy foods may undermine efforts made through dietary guidelines to promote healthier diets, especially concerning children, as television advertisements are the number one contributor to childhood obesity [52,53]. Media regulations that require transparency when advertising foods that exceed recommended sugar, salt, and fat intake could reduce unhealthy food consumption.

Furthermore, the temporal orientation of advertisements affects consumers’ health motivations, attitudes toward the advertisement, and the effectiveness of the advertisement in influencing purchase behavior and consumption patterns [54]. Advertisements for healthy foods were more frequent in the mornings, whereas advertisements for unhealthy foods and portrayed actors classified as overweight/obese were more frequent in the evenings, nights, and weekends. The temporal orientation of advertisements aligns with the depletion of self-regulation later in the day, as consumers are more health-conscious during the mornings but vulnerable to more indulgent and convenient food purchases and consumption during relaxation and leisure times [55]. Additionally, male actors were more prevalent on weekends than weekdays, suggesting potential gender bias in advertisements that should be further explored. Strategic timing in promoting specific foods and how foods are promoted highlights the need for policies that consider the foods being promoted and how they are being promoted to reduce their influence on unhealthy consumption patterns.

Advertisements used various strategies to increase the desirability and appeal of foods and beverages. Half of the advertisements used persuasive marketing techniques, more frequently when advertising unhealthy foods, consistent with findings from prior research in Ghana [56] and in high- and low-income countries [9]. Persuasive marketing techniques were more frequent for unhealthy foods, on weekends, and with male actors or those classified as overweight/obese. Persuasive marketing techniques have been found to increase the desirability of foods among Ghanaian children, who are susceptible to believing that consuming unhealthy food will help them resemble the celebrity advertising them [41,57]. Similarly, lower-income groups are more susceptible to discount strategies. The disproportional use of discount strategies in unhealthy food advertisements could increase consumption of these foods and contribute to an inequitable risk of developing diet-related NCDs. Instead, these techniques should be used to boost the appeal of healthy foods with normal-weight actors.

Advertisements shape consumer attitudes using normative, hedonic, and gain goal frames to influence how they feel, perceive benefits, or what they believe is socially acceptable behavior, respectively [42]. The higher prevalences of male actors in gain-framed advertisements and female actors in normative-framed advertisements likely reflect societal expectations, where females conform to idealized norms while men fulfill provider roles. Goal frames have been found to shape sustainable food choices in high-income countries [58–60]. Future research is needed to determine if using goal frames in mass media can be an effective large-scale intervention to promote healthy and sustainable food consumption in LMICs.

With globalization, the media’s promotion of Western ideals by portraying ultra-thin bodies is becoming more influential, challenging African norms and shifting Ghanaian body aspirations [36,61,62]. In West Africa, sociocultural norms have historically preferred larger body sizes for older females in urban settings, as a symbol of dignity, health, beauty, and wealth [24,25,27–31], whereas thinner body sizes have been preferred for males [25]. Television advertisements often reflect these shifting ideals, either reinforcing African stereotypes and stigma or promoting Western ideals [63,64]. The use of actors classified as underweight with a normative-frame advertisement suggests a cultural shift from the traditional preference for larger body sizes, still idealized among older women, to thinner bodies, idealized among adolescent girls and young women [26]. Future research is needed to understand the effect of actors’ body sizes on viewers in contexts where larger body sizes are historically preferred.

Exposure to ultrathin bodies in the media can lead to preoccupation with body image, potentially exacerbating eating disorders, disordered eating, body dysmorphia, and poor mental health. In Ghana, for example, youth have engaged in dieting behaviors to achieve the thinner bodies portrayed in the media [65]. When consumers see actors classified as underweight consuming unhealthy foods, they may associate these foods with slimmer body sizes and model the behavior of the actors with expectations of achieving a slimmer body size [66,67]. The use of unrealistic body sizes to advertise unhealthy foods sends conflicting messages that could influence the perceived healthiness of foods [67,68] and could contribute to disordered eating, obesity, and diet-related NCDs. Transitions in body ideals create vulnerabilities in self-image, leading to internalizing unrealistic beauty standards and dietary norms observed in the media. Internalizing body ideals fosters unfavorable comparisons and aspirations toward unattainable body sizes [69–71] and can lead to body dissatisfaction, low self-esteem, and disordered eating behaviors [72–75]. The global rise in eating disorders over the last 20 years [76], with nearly a quarter of children and adolescents experiencing disordered eating in 2023 [77], highlights the severity of unintended consequences of this marketing strategy. While evidence on eating disorders in Africa is scarce [78], it is likely rising, particularly among adolescent females.

Media messages and marketing techniques, particularly around body sizes, permeate every aspect of life, starting with children’s toys (e.g., Barbies), exerting pressure on society to conform to unrealistic body image ideals [79]. In addition to actors being primarily classified as underweight, these actors were disproportionately represented in advertisements promoting unhealthy foods, potentially influencing the perceived healthiness of foods [67,68]. When individuals observe certain stimuli, they tend to form associations through a cognitive process known as observational learning, as described by Social Cognitive Theory [34]. Viewers with more media literacy and nutritional knowledge can recognize these conflicting messages as marketing strategies, understanding that consuming foods high in sugars, fats, and salts generally leads to larger body sizes than the actors portrayed. There is a need for policies that enforce realistic nutrition and health messages and programs to increase media literacy to prevent mixed messages between health information and physical expectations. Social movements and policies in the fashion industry have made efforts to mitigate eating disorders caused by unrealistic body expectations [66,80,81]. Similar social movements and policies should be taken within the food industry to avoid extremely thin and overweight actors by primarily including normal-weight actors, avoid contributing to gender disparities in health, and maintain cultural values by tailoring advertisements to specific contexts.

### Strengths and limitations

This content analysis provides a comprehensive understanding of the marketing strategies and the physical characteristics of actors portrayed in food and beverage television advertisements in Ghana. To our knowledge, this is the first study to assess the body size of actors in food and beverage advertisements using Ettarh silhouettes and one of the few to have investigated the physical characteristics of actors. Although the 18 sex-specific silhouettes used to assess body size are validated and showed high inter-rater reliability [44,45], the tool remains subject to coder bias, as it relies on perception rather than objective body composition metrics that are impractical to assess in advertisements. The study is limited to quantifying the extent that body sizes appear in food and beverage advertisements. Future research is needed to assess the impact of these advertisements by better understanding who is exposed to these television advertisements, how they are perceived, and how they influence body image perceptions and food choice behaviors. The design was cross-sectional, and data were collected across a nationwide television channel. Future research should focus on changes over time and compare within and among countries based on nutrition transition phases, policies, and regulatory mechanisms. Although half of Ghanaians reported receiving health and nutrition education via television [56] and nearly two-thirds of households owned a television in 2018—higher in urban areas [82]—this study is limited by the decline in television exposure as social media exposure increases, particularly among youth, those with higher education and incomes, and urban residents. Our restricted analysis of television advertisements with actors found similar results to outdoor advertisements in the same study context and time [83], suggesting the generalizability of these findings across food and beverage marketing in Ghana. The characteristics of actors in television advertisements may serve as an accurate representation of actors portrayed to promote foods across various media platforms; however, future research on the actors portrayed in food and beverage advertisements through other social media platforms is needed.

## Conclusion

Television advertisements promoted foods and beverages high in sugar, salt, and fat, particularly during evenings and weekends, using persuasive marketing techniques, goal frames, and actors classified as underweight. These findings support the need for effective policies and practices that regulate food advertising and mass media to align with global nutrition targets and promote realistic messages for healthier populations.

## Supporting information

S1 Table A priori codebook that was used for Ghana’s television content analysis for food and beverage advertisements. (DOCX)
