# Supplementary material for: What, when, and how food and beverage are advertised on Ghanian television
Source: PLoS One. 2025 Jun 9;20(6):e0325730. doi: 10.1371/journal.pone.0325730 (PMC12148106; doi:10.1371/journal.pone.0325730)
Supplement: S1 Table — (DOCX) [file pone.0325730.s001.docx]

**S1 Table. A priori codebook that was used for Ghana’s television content analysis for food and beverage advertisements.**

| **Variable** | **Description** | **Response Format** |
| --- | --- | --- |
| daterecord | Date of recording | ddmmyyyy |
| timeslot | The time slot of Advertisement | 1 = 6:00-6:59  2 = 7:00-7:59  3 = 8:00-8:59  4 = 9:00-9:59  5 = 10:00-10:59  6 = 11:00-11:59  7 = 12:00-12:59  8 = 13:00-13:59  9 = 14:00-14:59  10 = 15:00-15:59  11 = 16:00-16:59  12 = 17:00-17:59  13 = 18:00-18:59  14 = 19:00-19:59  15 = 20:00-20:59  16 = 21:00-21:59  17 = 22:00-22:59  18 = 23:00-24:00 |
| foodcategory | Food product category*, select all that apply* | 1 = Breads, rice and rice products without added fat, sugar or salt, noodles (exclude fried), plain starch products (e.g., starch balls), plain biscuits and crackers  2 = Low sugar and high fiber breakfast cereals (<20g sugar /100g and >5g dietary fiber /100g)  3 = Fruits and fruit products without added fats, sugars, or salt (include fresh, tinned in natural juice, and dried, fruit juices containing ≥98% fruit)  4 = Vegetables and vegetable products without added fats, sugars, or salt (include fresh, tinned, and dried, plain seaweed)  5 = Milks and yoghurts (≤3g fat /100g), cheese (≤15g fat /100g) and their alternatives e.g., soy (include probiotic drinks)  6 = Meat and meat alternatives (include meat, poultry, fish, legumes, tofu, eggs and raw unsalted nuts)  7 = Oils high in mono- or polyunsaturated fats, (olive oil, sunflower oil, soyabean oil, plant-based margarines, and spreads), and low-fat savory sauces (<10g fat /100g)  8 = Low fat/salt meals (include frozen or packaged meals (≤6g saturated fat /serve, ≤900mg sodium /serve), soups (<2g fat /100g, exclude dehydrated), sandwiches, mixed salads, steamed buns (exclude sweet buns), wontons and dumplings not usually fried before consumption)  9 = Healthy Snacks – must be based on core foods (i.e., fruit, vegetables, grains, dairy, soy, meats or alternatives) and contain <600kJ / serve, <3g saturated fat /serve and <200mg sodium /serve  10 = Baby foods (exclude milk formulae)  11 = Bottled water (include unflavored mineral and soda waters)  12 = High sugar and/or low fiber breakfast cereals (>20g sugars /100g or <5g dietary fiber /100g)  13 = Flavored/fried instant rice and noodle products  14 = Sweet breads, cakes, muffins, sweet buns (e.g., lotus seed, custard, red bean), sweet biscuits (include egg rolls), sweet glutinous rice balls or cakes, high fat savory biscuits, pies and pastries, sweet sticky rice, or rice pudding.  15 = Meat and meat alternatives processed or preserved in salt (include Frankfurt’s, seafood sticks, jellyfish salad, tinned meats, and all preserved ready to eat meats, poultry, fish, tofu, and egg products)  16 = Sweet snack foods (include jelly, sugar-coated dried fruits or nuts, nut or seed-based bars and slices, sweet rice bars, and tinned fruit in syrup)  17 = Savory snack foods with added salt or fat (include chips, dried spicy peas, fruit chips, savory crisps, extruded snacks, popcorn (exclude plain), salted or coated nuts, other fried snacks (e.g., shrimp crackers))  18 = Fruit juice/drinks (<98% fruit)  19 = Full cream milks and yoghurts (>3g fat /100g) and cheese (>15g fat /100g, and high salt cheeses, (include haloumi and feta)) and their alternatives e.g., soy  20 = Ice cream, iced confection, and desserts  21 = Chocolate and candy (include marshmallows, sugar (all types), and chewing gums (exclude sugar free varieties))  22 = Fast food (not only healthier options advertised), e.g., burgers, fries, soft drinks (include if some but not all the foods/drinks advertised are healthier options)  23 = High fat/salt meals - frozen or packaged meals (>6g saturated fat /serve, >900mg sodium /serve) (include steamed buns (exclude sweet buns), wontons and dumplings usually fried before consumption)  24 = Other high fat/salt products (include meat/fish/bean pastes, XO sauce, butter and animal fats, high fat savory sauces (>10g fat /100), soups (>2g fat /100g and all dehydrated))  25 = Sugar sweetened drinks (include soft drinks, sweetened tea drinks, sports/electrolyte drinks, powdered flavor additions (e.g., Nesquik, sweetened tea or coffee powders))  26 = Alcohol  27 = Recipe additions (include soup cubes, oils, dried herbs and seasonings) Note: these foods are not usually consumed alone. They are added to flavor meals.  28 = Vitamin/mineral or other dietary supplements, and sugar-free chewing gum  29 = Tea and coffee (exclude sweetened powder-based teas or coffees)  30 = Baby and toddler milk formulae  31 = Fast food (only healthier options advertised), e.g., grilled chicken wrap, water, fruit slices  32 = Fast food (not only healthier options advertised), e.g., burgers, fries, soft drinks (include if some but not all the foods/drinks advertised are healthier options)  33 = Fast-food restaurant (NO foods or drinks advertised)  34 = Local restaurant  35 = Supermarkets (only core and healthy foods advertised)  36 = Supermarkets (not only core and healthy foods advertised)  37 = Supermarkets (NO foods or drinks advertised) |
| powered | Power of advertising | 1 = Strategy used  0 = No strategies used |
| powerstrategy | Power of advertising strategy*, select all that apply* | 1 = Cartoon/Company owned character (e.g., M&Ms)  2 = Licensed character (e.g., Dora the explorer)  3 = Amateur sportsperson (e.g., person playing a sport)  4 = Celebrity (non-sports) (e.g., Jamie Oliver)  5 = Movie tie-in (e.g., Shrek)  6 = Famous sportsperson/team (e.g., All Blacks)  7 = Non-sports/historical events/festivals (e.g. Christma Anzac Day)  8 = ‘For kids’ (e.g., image of a child, ‘great for school lunch,’ ‘for school lunchboxes’)  9 = Awards (e.g., Best Food Award 2014, award winning, number one best-selling’)  10 = Sports event |
| premium | Premium offers present | 1 = Premium offer used  0 = No premium offer used |
| premiumdesc | Premium offers type | 1 = Game and app downloads  2 = Contests  3 = Pay 2 take 3 or other  4 = 20% extra or other  5 = Limited edition  6 = Social charity  7 = Gift or collectable  8 = Price discount  9 = Loyalty programs |
| goalframe | Type of goal frame used*, select all that apply* | 1 = Normative  2 = Hedonic  3 = Gain |
| adhuman | Are there human(s) in the advert? | 1 = Yes  0 = No |
| num_longshot | How many long shot (whole body at least down to knees) shots are there in the advertisement? | 0 = None  1 = 1  2 = 2  3 = 3  4 = 4  5 ≥ 5 |
| childactor | Is/are there child actor(s) present in the advert? | 1 = Yes  0 = No |
| age_adolescent | Number of figure rated adolescents (ages 12-17) in advertisement | Response options: 0-5 |
| age_adult | Number of figure rated adults (18+) in advertisement | Response options: 0-5 |
| black | Number of figure rated people in advertisement whose race appears Black | Response options: 0-5 |
| white | Number of figure rated people in advertisement whose race appears White | Response options: 0-5 |
| asian | Number of figure rated people in advertisement whose race appears Asian (e.g., Middle Eastern, South or Southeast Asian) | Response options: 0-5 |
| indeterminant_race | Number of figure rated people in advertisement whose race cannot be determined | Response options: 0-5 |
| sex_male | Number of figure rated males in advertisement | Response options: 0-5 |
| sex_female | Number of figure rated females in advertisement | Response options: 0-5 |
| bodysize_male | Use rating scale to rate up to 5 models in the advertisement – rate first 5 models from left to right as they appear | Text, response options: 1-18 |
| bodysize_female | Use rating scale to rate up to 5 models in the advertisement – rate first 5 models from left to right as they appear | Text, response options: 1-18 |
